# Illinois State Medical Society

**Published:** 1858-05

**Authors:** 


### ILLINOIS STATE MEDICAL SOCIETY.

We hope it will be borne in mind that the Illinois State Medical Society will meet at the city of Rockford, Tuesday, June 1st, 1858. Our cotemporaries have very generally noticed the last year's transactions of the society, and we are proud of the commendation so universally awarded to the papers contained in them.

The profession in the State of Illinois is taking a position for respectability and intelligence, foremost in the march of scientific progress.

